# Rhabdomyolysis: A Rare Adverse Effect of Levetiracetam

**DOI:** 10.7759/cureus.2705

**Published:** 2018-05-29

**Authors:** Vaibhav Rastogi, Devina Singh, Babbaljeet Kaur, Pulkit Arora, Jaya P Gadikota

**Affiliations:** 1 Department of Medicine, University of Central Florida; 2 College of Medicine, University of Central Florida; 3 Family Medicine, North Florida Regional Medical Center; 4 Internal Medicine, North Florida Regional Medical Center

**Keywords:** levetiracetam, rhabdomyolysis, adverse effect

## Abstract

Levetiracetam is an anti-epileptic that works at the synapse and binds synapse vesicle protein 2A, thereby controlling the release of neurotransmitters. Its side effects mainly include somnolence, headache, fatigue, dizziness, vomiting, and behavioral alterations. Rhabdomyolysis is a rare adverse effect of levetiracetam. The underlying pathophysiology of this adverse effect is unknown. Our patient is a 42-year-old male who was brought to the hospital with a complaint of generalized tonic-clonic seizures and urinary incontinence. His symptoms were caused by hyponatremia. Levetiracetam was started for seizure prevention along with management for hyponatremia. His creatine phosphokinase levels increased on the third day of admission to 30,000 U/L. Four days after the discontinuation of levetiracetam and with the institution of supportive therapy, the patient’s rhabdomyolysis resolved.

## Introduction

Levetiracetam is a second-generation anti-epileptic that has been approved in the United States since 1999 for the treatment of generalized and partial seizures of multiple etiologies. Levetiracetam modulates the release of synaptic neurotransmitters by binding to synaptic vesicle protein 2A (SV2A) [1]. The adverse effects of levetiracetam are varied; the most common are somnolence (14%), headache (10%), fatigue (8%), accidental injury (8%), and dizziness (7%). Others include infection, flu syndrome, vomiting, and behavioral effects (depression, hostility, agitation, and anxiety) [2]. Rhabdomyolysis, as an adverse effect of levetiracetam, has not been well-defined in the literature; this is the sixth reported case. Here, we describe a case of levetiracetam-induced rhabdomyolysis.

## Case presentation

**Figure 1:**
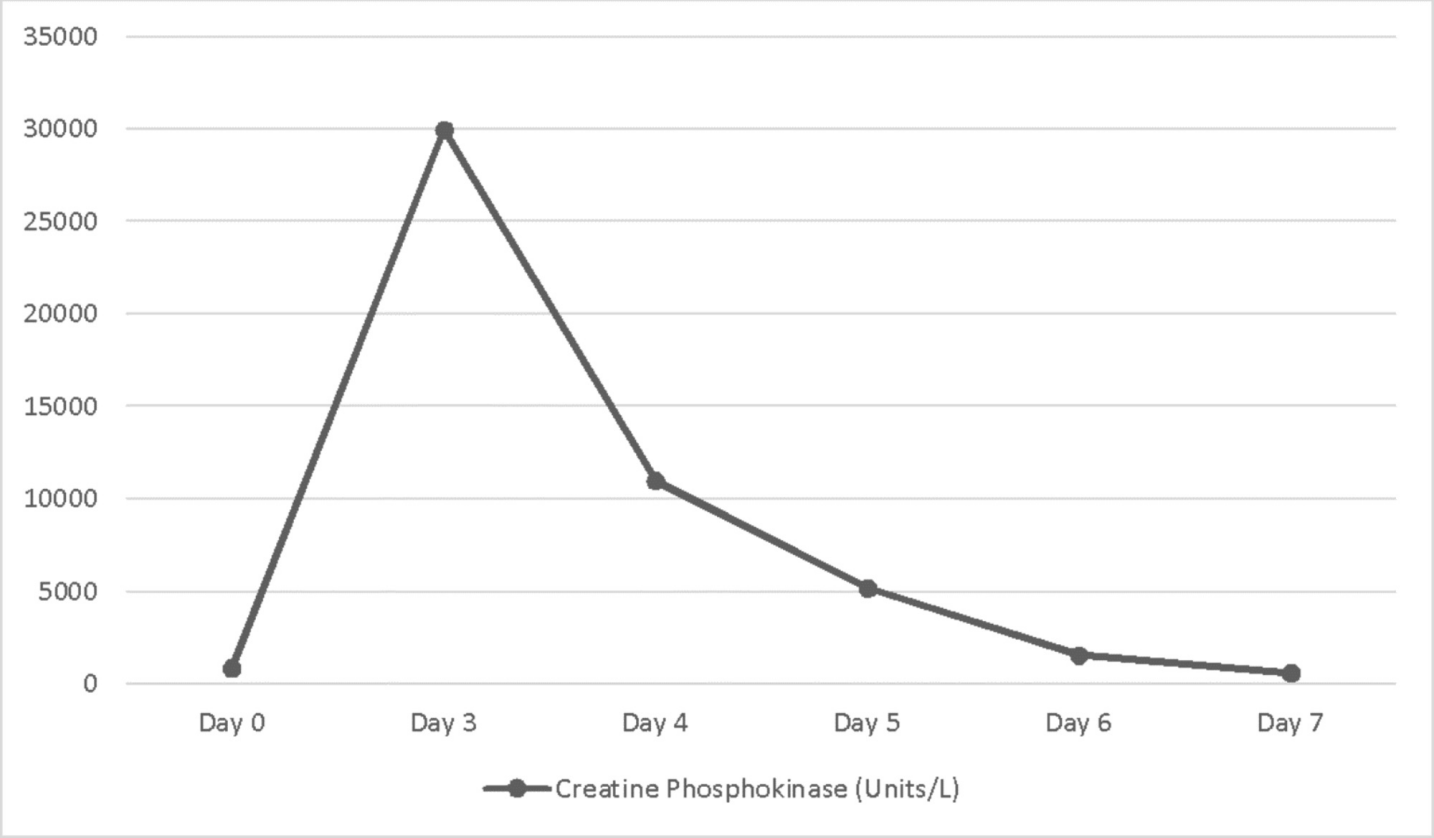
Trends in creatine phosphokinase levels during hospitalization

A 42-year-old male with a history of schizophrenia, hypertension, and bipolar disorder was brought to the hospital secondary to an episode of generalized body shaking and urinary incontinence. Vital signs were stable (temperature 95.8 F, heart rate 98, blood pressure 150/87 mmHg, respiratory rate 16, and oxygen saturation 97% on room air). The physical examination, except the neurological exam, was unremarkable. The patient was extremely lethargic and was alert only to place. He followed basic commands and did not have any significant cranial nerve, motor, or sensory deficits. Blood work was significant for sodium of 106 mEq/L with serum osmolality of 226 U/L and creatine phosphokinase (CPK) of 835 U/L. The sodium was corrected cautiously with a rate of 8-10 mEq/L/24 hours with intravenous normal saline, desmopressin, and fluid restriction (1200ml/24 hours). The patient received two 500 mg doses of levetiracetam. He was continued on intravenous levetiracetam 750 mg every 12 hours. His home medications, including benztropine, risperidone, and trazodone, were initiated. On the third day of admission, the patient`s CPK levels were elevated to more than 30,000 U/L. His creatinine increased from 1.01 to 1.51 mg/dl. Urine myoglobin was also elevated. He was diagnosed to have an acute kidney injury and rhabdomyolysis. Levetiracetam was immediately discontinued, as it was considered to be the possible etiology. The patient was treated with aggressive intravenous fluid hydration (normal saline) for rhabdomyolysis and for the improvement of renal function. A gradual improvement of CPK was noted (Figure 1) accompanied by an improvement in renal function as well as in the overall clinical condition of the patient.

## Discussion

Rhabdomyolysis is a condition that is characterized by the destruction of skeletal muscle and the spillage of its contents into the bloodstream. These include mainly sarcoplasmic proteins (CPK, lactate dehydrogenase), myoglobin, and electrolytes [3]. The etiology of rhabdomyolysis is diverse, comprising trauma, genetic disorders, strenuous exercise, seizures, infections, electrolyte abnormalities, and medications. Clinically, it manifests with a classic triad of myalgia, fatigue, and dark-colored urine [3]. The elevation of CPK is a hallmark finding seen in this patient subset; lactate dehydrogenase elevation and myoglobinuria are also commonly observed. Acute kidney injury is an important complication. Early recognition and treatment with fluids are essential to prevent kidney injury. Electrolyte management is also vital for prognosis [3].

Rhabdomyolysis has been documented as a rare adverse effect of levetiracetam, and until now, only five cases have been reported in the literature. Three of them were pediatric patients and the other two patients were in the late 20s [1,4-7]. Our patient is a 42-year-old male who received levetiracetam for seizure treatment and prophylaxis. Multiple factors could predispose our patient to rhabdomyolysis, including seizures, hyponatremia correction, and concomitant medications (risperidone). Brigo et al., in their review, suggested that seizures usually cause a slight elevation of CPK (< 180 U/L) and the peaks are noted at 36-40 hours [8]. In our patient, the CPK elevations were significantly higher (> 30,000 U/L) and were noted at 72 hours after admission. A review by Kashiura et al. noted a correlation between higher sodium correction rates (> 1 mEq/L/hour) and rhabdomyolysis [9]. However, the sodium correction rate in our case report was 8-10 mEq/L/24 hours, which equates to <0.5 mEq/L/hour, which makes it very less likely to have rhabdomyolysis as a result of serum sodium correction. Drastic elevations in the levels of CPK after the initiation of levetiracetam and a decrease in CPK levels after the withdrawal of the drug indicates that it was likely levetiracetam that caused the rhabdomyolysis. Even though causality can’t be completely established, as it would have been unethical to re-expose the patient to levetiracetam, but with the help of the adverse drug reaction probability scale developed by Naranjo et al. [10], we can deduce that rhabdomyolysis can probably be caused by levetiracetam.

## Conclusions

Rhabdomyolysis is a rare adverse effect of levetiracetam. The underlying pathophysiological mechanism for this adverse effect still remains unknown. Further studies are required to investigate the effect of levetiracetam on muscles, especially skeletal muscle.
